# Inactivation of cAMP receptor protein (MSMEG_6189) increases isoniazid susceptibility in *Mycobacterium smegmatis* via altered oxidative phosphorylation, elevated ROS production, and loss of *ahpC* expression

**DOI:** 10.1128/jb.00429-25

**Published:** 2025-11-24

**Authors:** Narin Kim, Yuna Oh, Jeong-Il Oh

**Affiliations:** 1 Department of Integrated Biological Science, Pusan National University, Busan, South Korea; 2 Microbiological Resource Research Institute, Pusan National University, Busan, South Korea; The Ohio State University, Columbus, Ohio, USA

**Keywords:** AhpC, cAMP receptor protein, electron transport chain, isoniazid, KatG, *Mycobacterium*, ROS

## Abstract

Isoniazid (INH) is a first-line drug for treating drug-susceptible tuberculosis. The genome of *Mycobacterium smegmatis* encodes two cAMP receptor proteins (Crp), MSMEG_6189 (Crp1) and MSMEG_0539 (Crp2). The deletion of *crp1* markedly increased susceptibility to INH, whereas the deletion of *crp2* had no effect. In contrast, susceptibility to rifampicin was unchanged in the Δ*crp1* mutant. Gene expression analysis revealed strong upregulation of *katG1*, which encodes the INH-activating catalase-peroxidase, and an almost complete abolishment of *ahpC* expression, which encodes alkyl hydroperoxide reductase and contributes to INH resistance, in the Δ*crp1* mutant. The mutant also exhibited elevated intracellular reactive oxygen species (ROS) levels and reduced respiration. These elevated ROS levels likely resulted from alterations in the respiratory electron transport chain, including reduced levels of the cytochrome *bcc*_1_ complex, increased expression of the *nuo* and *sdh1* operons (encoding type I NADH dehydrogenase and succinate dehydrogenase, respectively), and the loss of *bd* quinol oxidase induction under conditions of diminished *bcc*_1_ complex levels. ROS accumulation, in turn, appears to inactivate the FurA repressor, leading to the induction of *katG1* expression. Collectively, these findings suggest that the loss of Crp1 increases INH susceptibility through multiple mechanisms that promote INH activation, including ROS-mediated induction of *katG1* and increased intracellular ROS levels. Furthermore, the absence of *ahpC* expression in the Δ*crp1* mutant further contributes to its increased INH susceptibility.

**IMPORTANCE:**

This study revealed that the cAMP receptor protein, Crp1, plays a role in controlling key genes involved in both INH activation (*katG1*) and resistance (*ahpC*) in *Mycobacterium smegmatis*. Inactivation of Crp1 was shown to cause an increase in cellular ROS levels, which likely results from a bottleneck of electron flow at the cytochrome *bcc*_1_ complex in the electron transport chain and the abolishment of *bd* quinol oxidase induction under conditions of reduced *bcc1* complex levels. The resulting increase in ROS, combined with the simultaneous loss of *ahpC* expression, renders the bacterium significantly more vulnerable to INH. These findings provide deeper insights into the interplay among oxidative phosphorylation, ROS generation, and INH susceptibility.

## INTRODUCTION

Tuberculosis (TB), the leading cause of death from a single pathogenic bacterium, is an infectious disease that is caused by *Mycobacterium tuberculosis* infection (1). The first-line TB drugs standardly used to treat drug-susceptible TB include isoniazid (isonicotinic acid hydrazide, INH), rifampicin (RIF), pyrazinamide, and ethambutol (2). INH is a prodrug that is activated by the KatG catalase-peroxidase (3). The enzyme’s peroxidatic activity catalyzes the oxidation of INH, leading to the formation of an isonicotinoyl radical (4). The radical then interacts with NAD^+^, forming an INH-NAD adduct that inhibits the activity of NADH-dependent enoyl acyl carrier protein (ACP) reductase (InhA), an enzyme involved in the synthesis of mycolic acids of the mycobacterial envelope (5). Superoxide radicals, hydrogen peroxide (H_2_O_2_), and simple alkyl hydroperoxides have been proposed as potential oxidants that promote the KatG-mediated conversion of INH to the isonicotinoyl radical (6–9).

At least 80% of INH-resistant strains are estimated to carry either mutations in *katG* or *inhA* (10). The *ahpC* gene encodes alkyl hydroperoxide reductase that reduces H_2_O_2_ and organic peroxides together with the AhpD peroxiredoxin reductase, dihydrolipoamide succinyltransferase (SucB), and dihydrolipoamide dehydrogenase (Lpd) using NADH as the reductant (11–14). Overexpression of *ahpC* has been shown to increase INH resistance in mycobacteria, whereas the inactivation of *ahpC* in *M. smegmatis* results in increased INH susceptibility (15, 16). In addition, several other genes involved in altered INH tolerance in mycobacteria have been identified, including *mspA*, *rbpA*, *ndh*, *ctaC*, *mdp1*, and *cydA*. The *mspA* gene codes for a porin protein that is predominantly expressed in mycobacteria. When *mspA* from *M. smegmatis* was overexpressed in *M. tuberculosis* and *Mycobacterium bovis*, the susceptibility of both bacterial species to INH was increased (17). RbpA, an RNA polymerase-binding protein, was shown to be related to INH tolerance in mycobacteria (18). The intracellular NAD^+^/NADH ratio has been suggested to affect INH tolerance in mycobacteria. Reduced NAD^+^ levels may limit the formation of the INH-NAD adducts, while increased NADH levels could protect InhA from inhibition by the INH-NAD adduct through competitive binding (19, 20). In line with this suggestion, mutations in the *ndh* gene, which encodes the major NADH dehydrogenase (NDH) of the respiratory electron transport chain (ETC), have been found in some INH-resistant *M. tuberculosis* isolates (19, 21). In addition, the inactivation of the major terminal oxidase of the respiratory ETC, the *aa*_3_ cytochrome *c* oxidase, through deletion mutations in *ctaC* or *ctaE*, has been shown to increase INH tolerance in *M. smegmatis* (22, 23). Mycobacterial DNA-binding protein 1 (MDP1) is a histone-like protein conserved among mycobacteria. Its inactivation in *M. smegmatis* has been shown to increase *katG* expression, leading to greater susceptibility to INH (24). The *cydA* gene encodes the catalytic subunit of the cytochrome *bd* quinol oxidase. The inactivation of *cydA* by transposon mutagenesis has been shown to increase INH susceptibility in *M. smegmatis* (25). Conversely, increased expression of the *bd* quinol oxidase has been reported to protect *M. bovis* BCG from INH-mediated killing (26).

cAMP is a universal secondary messenger involved in the intracellular signal transduction in both prokaryotes and eukaryotes. The cAMP receptor protein (Crp) is a global transcription factor with a homodimeric quaternary structure. It regulates the expressions of numerous genes involved in various metabolic and cellular processes in prokaryotes, including carbon utilization, respiration, virulence, reactivation of nonreplicating dormant cells, and stress responses (27–32). Unlike *M. tuberculosis*, which has a single *crp* gene, *M. smegmatis* possesses two *crp* genes, *MSMEG_6189* (*crp1*) and *MSMEG_0539* (*crp2*). Crp1 shares 78% amino acid sequence identity with Crp2 (33, 34). Recently, Crp1 was identified as the major Crp in *M. smegmatis*, based on the relative cellular protein levels of Crp1 and Crp2. The inability to generate a *crp1crp2* double-knockout mutant suggests that Crp may be essential for the survival or growth of *M. smegmatis* (35).

Among the genes involved in INH resistance, the regulation of *katG* and the *ahpCD* operon has been well studied in *M. smegmatis* (36, 37). *M. smegmatis* possesses two *katG* genes, *katG1* (*MSMEG_6384*) and *katG2* (*MSMEG_3461*), which are located within the *furA1-katG1* and *furA2-katG2* operons, respectively. The *furA1* (*MSMEG_6383*) and *furA2* (*MSMEG_3460*) genes encode the peroxide-sensitive regulators of the PerR family. In addition, another *furA* gene, *furA3* (*MSMEG_6253*), is present at a separate locus in the *M. smegmatis* genome. The three FurA paralogs have been shown to be functionally redundant in repressing the expressions of *katG1* and *ahpC*. FurA1 and FurA3 form the homodimeric quaternary structures, which are typical of most Fur (ferric uptake repressor) and PerR homologs, while FurA2 exists as a monomer. Upon exposure to hydrogen peroxide, FurA proteins are inactivated by the metal-catalyzed oxidation of a conserved histidine residue, resulting in derepression of *katG1* and *ahpC*. Peroxide treatment of *M. smegmatis* induces the expressions of *katG1* and *ahpC*, whereas the expression of *katG2*, which is regulated exclusively by monomeric FurA2, remains uninduced under these stress conditions (36, 37). In addition to being negatively regulated by the FurA paralogs, the expression of *ahpC* is positively regulated by Crp1 in *M. smegmatis* (37).

Mycobacteria possess a branched respiratory ETC that is terminated with two distinct terminal oxidases (38, 39). One pathway consists of the *aa*_3_-type cytochrome *c* oxidase associated with the cytochrome *bcc*_1_ complex, whereas the alternate route ends with the *bd*-type quinol oxidase (40–42). In *M. smegmatis* and *M. tuberculosis* grown under aerobic conditions, the *aa*_3_ cytochrome *c* oxidase functions as the major terminal oxidase, and its activity is, therefore, required for optimal aerobic growth (38, 39, 43, 44). The *bd* quinol oxidase, which has a high affinity for oxygen, becomes particularly important when the function of the *bcc*_1_-*aa*_3_ branch of the ETC is impaired, such as under hypoxic conditions (35, 38, 45, 46). Its expression is markedly induced under respiration-inhibitory conditions in a Crp1-dependent manner (30, 35). In mycobacteria, the major entry point of electrons into the ETC is the transfer from NADH to menaquinone. Mycobacteria possess two types of NDHs, the proton-pumping type I NDH and the nonproton-pumping type II NDH (47). Type I NDH, which is functionally equivalent to the mitochondrial complex I, consists of 14 subunits (NuoA–NuoN; MSMEG_2050–MSMEG_2063). In contrast, type II NDH is composed of a single polypeptide (Ndh; MSMEG_3621) and serves as the major NDH in the respiratory ETC of *M. smegmatis* (19, 20). Electrons can also enter the ETC through transfer from succinate to menaquinone, catalyzed by succinate dehydrogenase (SDH). *M. smegmatis* contains two operons that encode SDH: *sdh1CAB* (*MSMEG_0420-MSMEG_0417*) and *sdh2CDAB* (*MSMEG_1672-MSMEG_1669*). SDH1 is a type F SDH composed of three subunits (SdhA, SdhB, and SdhC), whereas SDH2 is a type A SDH composed of five subunits (SdhA, SdhB, SdhC, SdhD, and SdhF) (48–50).

In this study, we investigated whether Crp inactivation affects INH and RIF susceptibility in *M. smegmatis*. We found that the inactivation of the *crp1* gene, which encodes the major Crp, significantly increases INH susceptibility. This increased susceptibility is attributable to elevated cellular ROS levels, resulting from an altered functionality of the respiratory ETC, in combination with the loss of *ahpC* expression.

## RESULTS

### Crp1 inactivation increases INH susceptibility in *M. smegmatis*

**Fig 1:**
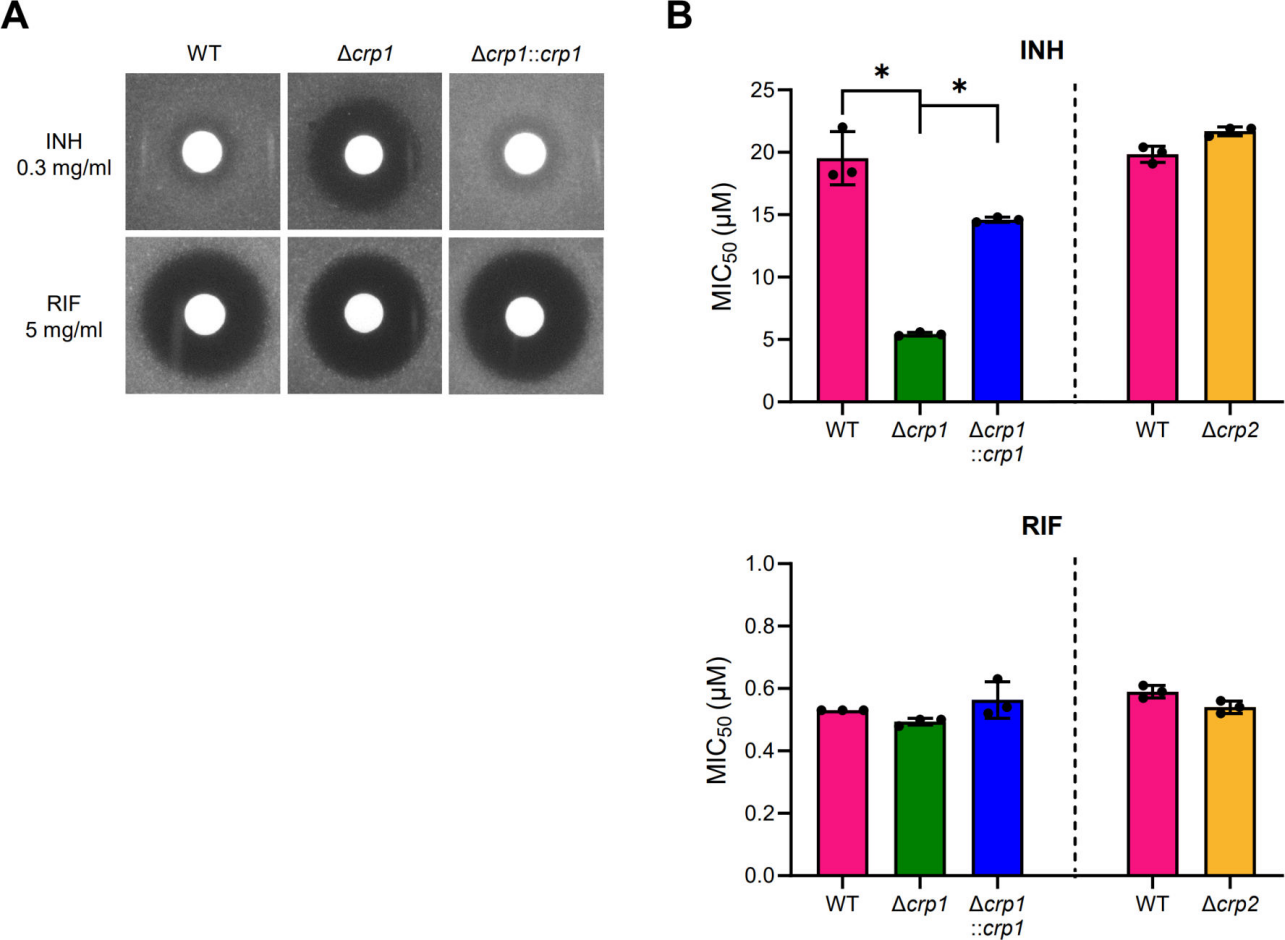
Susceptibility of the WT and Δ*crp1* strains of *M. smegmatis* to INH and RIF. (**A**) Zone inhibition assay of the WT and Δ*crp1* mutant strains carrying the empty vector pMV306. For complementation, the intact *crp1* gene was introduced into the Δ*crp1* mutant via pMV306crp, resulting in the Δ*crp1::crp1* strain. The diameter of the growth-inhibition zones around the disks reflects antibiotic susceptibility. (**B**) MIC_50_ of INH and RIF for the WT, Δ*crp1*, and Δ*crp2* strains of *M. smegmatis* carrying pMV306crp, as well as the complemented Δ*crp1::crp1* strain. Due to its slower growth, the Δ*crp1* mutant harboring pMV306 was cultured for 42 h, while all other strains were cultured for 24 h following antibiotic treatment. All values represent the averages of three biological replicates, with error bars indicating standard deviations. *P < 0.05.

To examine the effect of *crp1* inactivation on INH and RIF susceptibility in *M. smegmatis*, a zone inhibition assay was performed using the wild-type (WT) and Δ*crp1* mutant strains of *M. smegmatis* carrying the empty vector pMV306. The Δ*crp1* mutant harboring pMV306crp, which contains an intact *crp1* gene, was also included for the complementation test. The assay revealed that the Δ*crp1* mutant carrying pMV306 exhibited significantly increased susceptibility to INH compared to the WT strain with pMV306. In contrast, the mutant showed no difference in RIF susceptibility relative to the WT strain. Introduction of pMV306crp into the Δ*crp1* mutant reduced the size of the INH-inhibitory zone to a level comparable to that of the WT strain carrying pMV306, indicating that the increased INH susceptibility of the mutant is due to the *crp1* deletion (Fig. 1A). To quantitatively assess antibiotic susceptibility, we determined the MIC_50_ values of the WT and Δ*crp1* mutant strains for both INH and RIF (Fig. 1B). The Δ*crp2* mutant was also included in this analysis. Consistent with the results of the zone inhibition assay, the Δ*crp1* mutant carrying pMV306 showed a significantly reduced MIC_50_ for INH compared to the WT strain with pMV306. The mutant complemented with pMV306crp exhibited a restored MIC_50_, although it did not reach the level observed in the WT strain carrying pMV306. This partial complementation is likely due to lower expression of *crp1* from pMV306crp compared to its native chromosomal promoter. In contrast to *crp1*, the inactivation of *crp2* did not affect susceptibility to either INH or RIF, indicating that Crp2 is not involved in INH and RIF susceptibility.

### Inactivation of Crp1 leads to increased *katG1* expression and abolishment of *ahpC* expression

**Fig 2:**
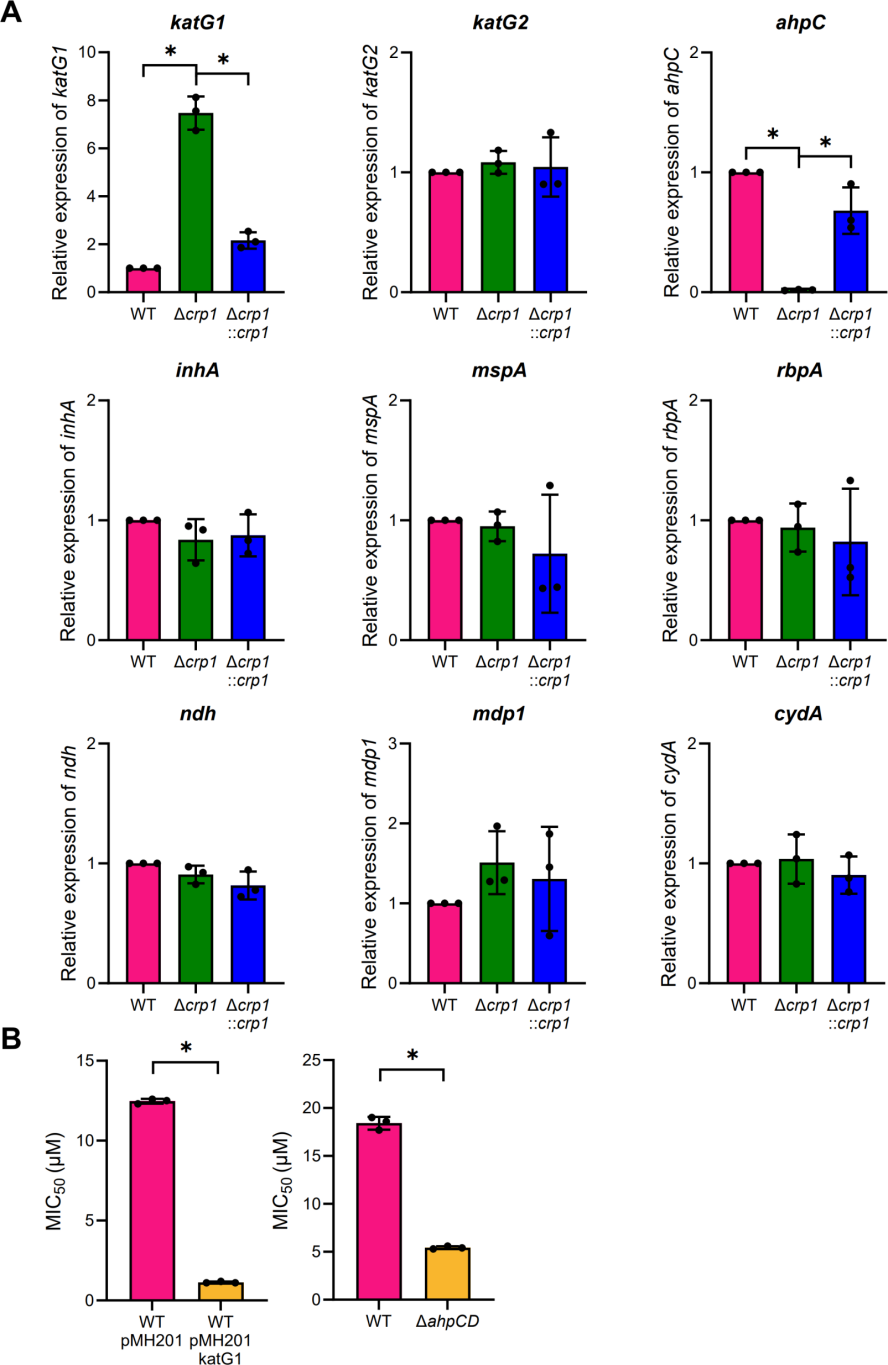
Expression of INH resistance/tolerance-related genes in the WT and Δ*crp1* strains of *M. smegmatis* (**A**) qRT-PCR analysis of *katG1, katG2, ahpC*, *inhA, mspA, rbpA, ndh, mdp1*, and *cydA* in the WT and Δ*crp1* strains harboring pMV306, as well as in the complemented Δ*crp1::crp1* strain. Gene expression was normalized to *sigA*. The expression level in the WT strain was set to 1, and relative expression values were calculated for the other strains. (**B**) Effect of *katG1* overexpression or *ahpC* inactivation on INH susceptibility, measured as MIC_50_. The WT strain overexpressing *katG1* (WT/pMH201katG1) and the Δ*ahpCD* mutant were compared with the WT strain carrying the empty vector (WT/pMH201) and WT without vector, respectively. All values represent the averages of three biological replicates, with error bars indicating standard deviations. *P < 0.05.

To ascertain the mechanism underlying the increased INH susceptibility of the Δ*crp1* mutant, we measured the expression levels of genes known to associate with INH resistance and susceptibility in mycobacteria using qRT-PCR. The analysis was conducted using the WT and Δ*crp1* strains carrying pMV306, as well as the Δ*crp1* mutant with pMV306crp (Fig. 2A). The analysis revealed that *katG1* expression was increased by 7.5-fold in the Δ*crp1* mutant with pMV306 compared to the WT strain with the same vector. Introduction of pMV306crp into the mutant reduced the expression of *katG1*. In contrast, *ahpC* expression was nearly abolished in the Δ*crp1* mutant, and complementation with pMV306crp restored its expression. This Crp1-dependent expression of *ahpC* is consistent with our previous report demonstrating the positive regulation of *ahpC* by Crp1 in *M. smegmatis* (37). Aside from *katG1* and *ahpC*, no significant changes in gene expressions were observed in the Δ*crp1* mutant relative to the WT strain for the other tested genes (*katG2*, *inhA*, *mspA*, *rbpA*, *ndh*, *mdp1*, and *cydA*).

To examine whether *katG1* upregulation or the loss of *ahpC* expression contributes to the increased INH susceptibility of the Δ*crp1* mutant, we determined the MIC_50_ for INH using the WT strain carrying the *katG1*-overexpressing integration plasmid pMH201katG1 and the Δ*ahpCD* mutant in which the *ahpCD* operon is deleted. As shown in Fig. 2B, the WT strain carrying pMH201katG1 exhibited a much lower MIC_50_ compared to the WT strain carrying the empty vector pMH201. Similarly, the deletion of the *ahpCD* operon led to a significant decrease in MIC_50_. Taken together, our results suggest that both the upregulation of *katG1* and the loss of *ahpC* expression in the Δ*crp1* mutant lead to its increased INH susceptibility.

**Fig 3:**
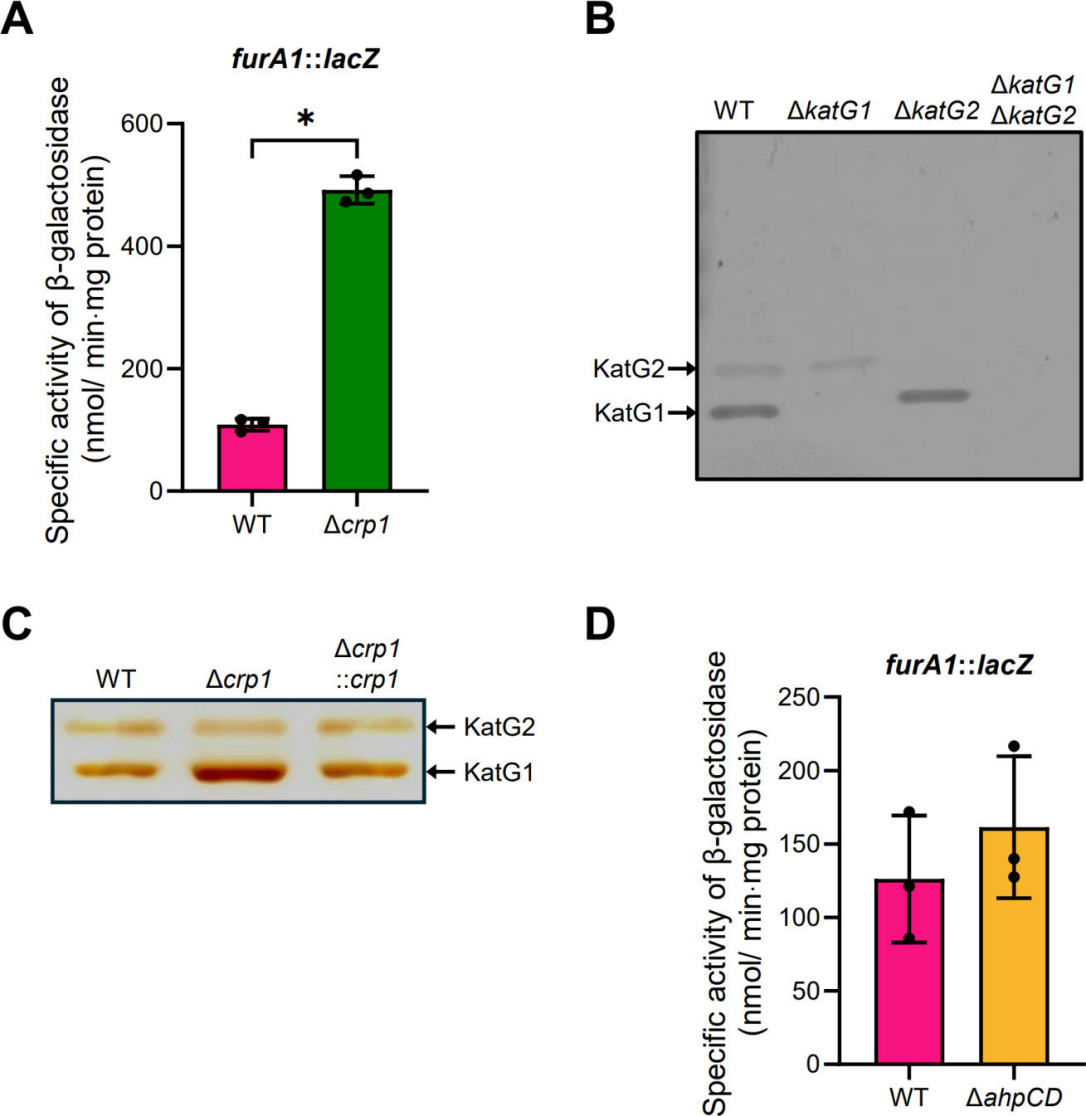
Validation of increased *katG1* expression in the Δ*crp1* mutant of *M. smegmatis* and effect of *ahpCD* deletion on *katG1* expression. (**A**) Promoter activity of the *furA1-katG1* operon in WT and Δ*crp1* strains. (**B**) Identification of the KatG1 and KatG2 bands by peroxidase activity staining using the WT, Δ*katG1*, Δ*katG2,* and Δ*katG1*Δ*katG2* strains. (**C**) Peroxidase activity staining of the WT and Δ*crp1* strains. For panels B and C, 25 µg of cell-free crude extracts was analyzed by native PAGE. (**D**) Promoter activity of the *furA1-katG1* operon in the WT and Δ*ahpCD* strains. For panels A and D, β-galactosidase activity was measured in strains carrying the *furA1::lacZ* transcriptional fusion plasmid pEMfurA1 grown aerobically to an OD_600_ of 0.45–0.5. All values represent the averages of three biological replicates, with error bars indicating standard deviations. *P < 0.05.

To confirm the qRT-PCR result showing upregulation of *katG1* expression in the Δ*crp1* mutant, we assessed the expression levels of the *furA1-katG1* operon in the WT and Δ*crp1* mutant using the *furA1::lacZ* transcriptional fusion plasmid pEMfurA1. As shown in Fig. 3A, the promoter assay demonstrated that *furA1* expression was increased by 4.5-fold in the Δ*crp1* mutant compared to the WT strain. Next, we examined *katG1* expression at the protein level in the WT and Δ*crp1* mutant strains using peroxidase activity staining. To identify the bands corresponding to KatG1 and KatG2 in the activity staining gel, crude extracts from the WT, Δ*katG1*, Δ*katG2*, and Δ*katG1*Δ*katG2* strains were subjected to peroxidase activity staining (Fig. 3B). In the activity staining gel, two bands were observed in the WT strain. The lower thick band was absent in the Δ*katG1* mutant, while the upper band disappeared in the Δ*katG2* mutant, indicating that the lower band represents KatG1 and that KatG1 is the major catalase-peroxidase in the WT strain grown aerobically in 7H9-glucose medium. Peroxidase activity staining in Fig. 3C clearly showed that KatG1 protein levels were increased in the Δ*crp1* mutant carrying pMV306 relative to the WT strain with the same vector. Introduction of pMV306crp into the Δ*crp1* mutant reduced KatG1 levels compared to those observed for the WT strain. In contrast to KatG1, KatG2 protein levels remained unchanged in the Δ*crp1* mutant compared to the WT strain, which is consistent with the qRT-PCR result. These results further support the conclusion that *katG1* expression is upregulated in the Δ*crp1* mutant. It is possible that the increased *katG1* expression observed in the Δ*crp1* mutant results from a compensatory effect caused by alkyl hydroperoxide reductase (AhpC) deficiency. To test this possibility, we measured the expression levels of the *furA1-katG1* operon in the WT and Δ*ahpCD* strains using pEMfurA1 (Fig. 3D). Expression of *furA1* was only marginally higher in the Δ*ahpCD* mutant than in the WT strain, indicating that the elevated *katG1* expression in the Δ*crp1* mutant is not due to the abolishment of *ahpC* expression. These results further suggest that the increased susceptibility of the Δ*ahpCD* mutant to INH is not due to KatG overproduction.

### Crp1 inactivation increases intracellular ROS levels in *M. smegmatis*

**Fig 4:**
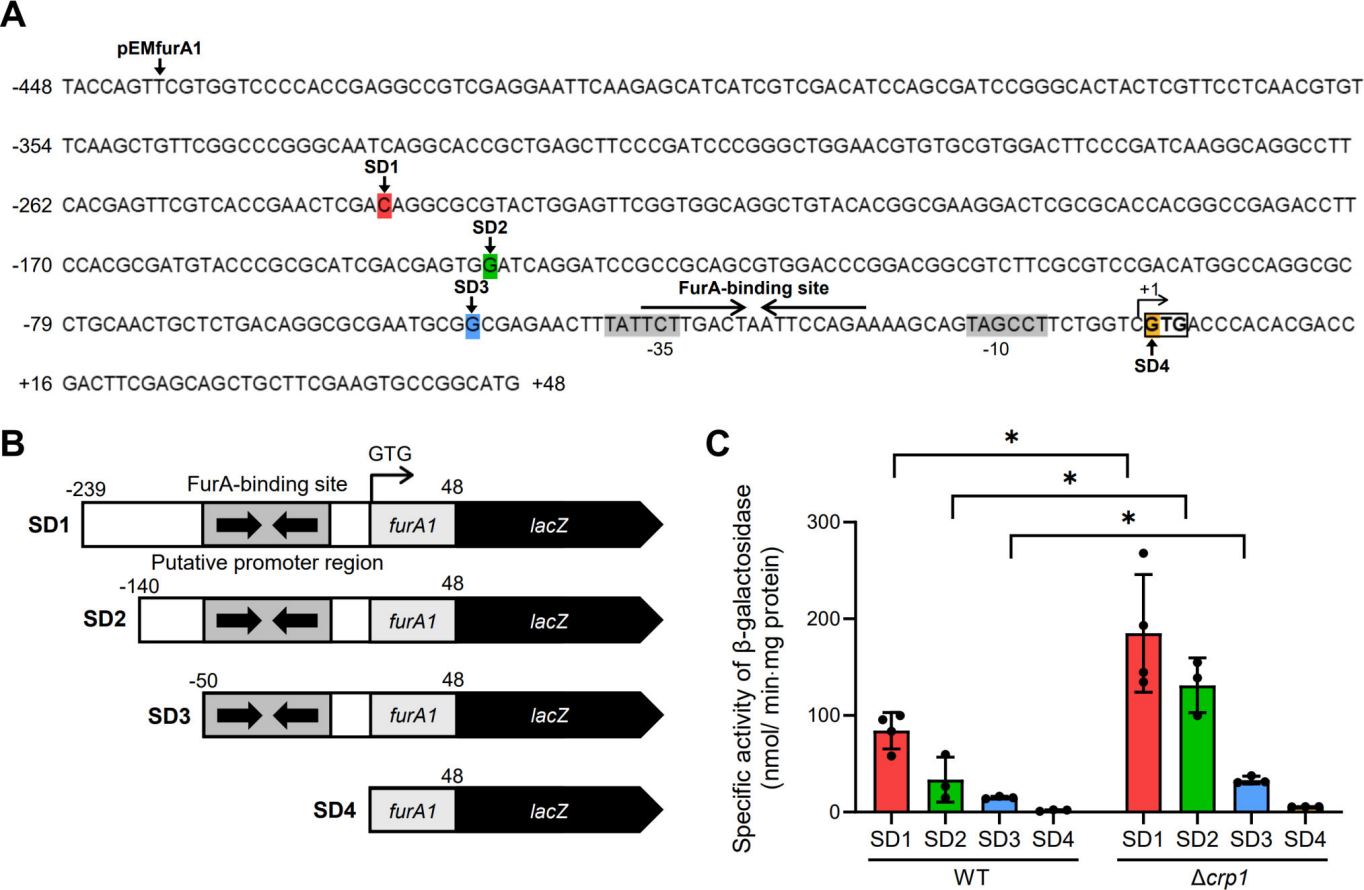
Identification of the *furA1* upstream region responsible for *katG1* induction in the Δ*crp1* mutant. (**A**) Upstream sequence of the *furA1-katG1* operon. The start codon (GTG) of *furA1* is boxed, and the transcription start point (+1) is indicated by an arrow (transcription of the operon produces a leaderless mRNA). The −10 and −35 promoter elements are shaded, and the FurA-binding site is marked by head-to-head arrows. The first nucleotides of the regions cloned in pEMfurA1 and the serially truncated *furA1::lacZ* transcriptional fusion plasmids (pEMSD1-4) are indicated by colored shading and arrows labeled as SD1, SD2, SD3, and SD4. All constructs contain the 5′ end of *furA1* extending 48 bp downstream of the start codon. (**B**) Schematic diagrams of pEMfurA1 and truncated transcriptional fusion plasmids. Gray boxes represent promoter regions, and the overlapping FurA-binding site is marked by head-to-head arrows. Numbers indicate the lengths (bp) of the *furA1* upstream regions and 5′ end included in each fusion. (**C**) β-galactosidase activity of WT and Δ*crp1* strains carrying pEMSD1-4. Strains were grown aerobically in 7H9-glucose medium to an OD_600_ of 0.45–0.5, and crude extracts were assayed for activity. All values represent the averages of three biological replicates except that four biological replicates were used for the WT and Δ*crp1* strains carrying pEMSD1 in panel C. Error bars indicate standard deviations. *P < 0.05.

**Fig 5:**
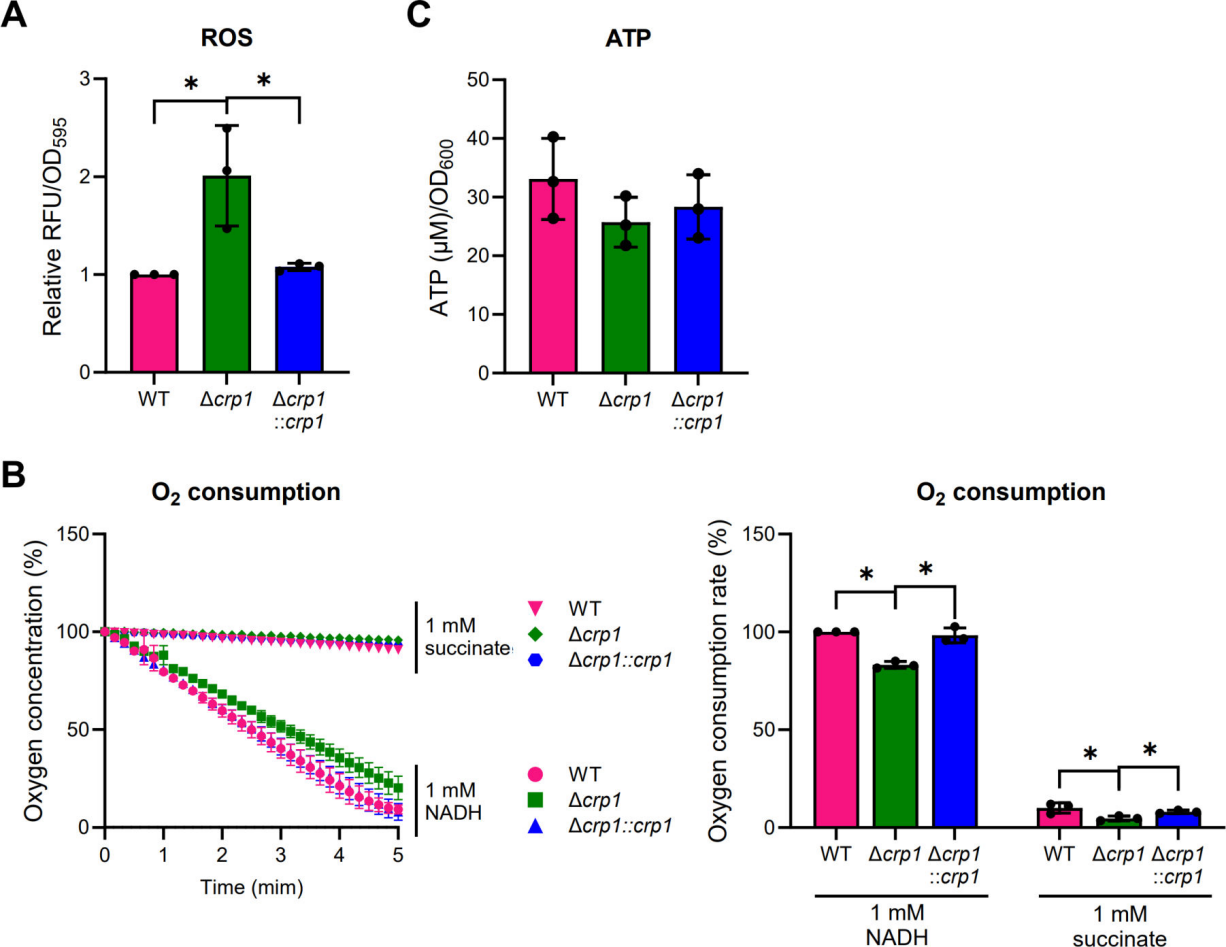
Intracellular ROS and ATP levels and oxygen consumption rates in the WT and Δ*crp1* strains of *M. smegmatis*. (**A**) Intracellular ROS levels in the WT and Δ*crp1* strains and the complemented Δ*crp1* mutant carrying pMV306crp (Δ*crp1::crp1*), quantified by DCF-DA staining in cultures grown aerobically to an OD_600_ of 0.45–0.5. ROS levels in the WT strain were set to 1, and the relative values were calculated for the other strains. (**B**) Oxygen consumption rates of the WT and Δ*crp1* strains with pMV306 and the Δ*crp1::crp1* strain. Membrane fractions (500 µg) were analyzed using a Clark-type electrode with NADH or succinate as electron donors. Left: time-dependent O₂ depletion after addition of the substrate. Right: oxygen consumption rates of the WT strain with NADH as the electron donor were set to 100, and the relative values were calculated for the others. (**C**) Intracellular ATP levels in the WT and Δ*crp1* strains with pMV306, and the Δ*crp1::crp1* strain, measured using cultures grown aerobically to an OD_600_ of 0.45–0.5. All values are the averages of three biological replicates. Error bars indicate standard deviations. *P < 0.05.

To delineate the upstream control region of *furA1* responsible for upregulation of the *furA1-katG1* operon in the Δ*crp1* mutant, we constructed a series of *furA1::lacZ* transcriptional fusion plasmids, each carrying varying lengths of the *furA* upstream region (239, 140, 50, and 0 bp) and a 48 bp 5′ portion of *furA1* (pEMSD1, pEMSD2, pEMSD3, and pEMSD4) (Fig. 4A and B). Using these plasmids, we measured the expression levels of *furA1* in both WT and Δ*crp1* strains (Fig. 4C). The expression levels of *furA1* were increased several-fold in the Δ*crp1* mutant carrying pEMSD1, pEMSD2, or pEMSD3 compared to the WT strain with the same plasmid. In contrast, *furA1* expression was not detected in either the WT or Δ*crp1* strain with pEMSD4 lacking the *furA1* promoter. These results indicate that the DNA region comprising the 50 bp upstream sequence of *furA1* and the 48 bp 5′ portion of *furA1* contains a *cis*-regulatory element involved in upregulation of *furA1* in the Δ*crp1* mutant. The 50 bp upstream sequence of *furA1* in pEMSD3 contains the *furA1* promoter and an overlapping FurA-binding sequence (Fig. 4A). A search of the 50 bp upstream sequence of *furA1* and the 48 bp 5′ portion of *furA1* did not reveal any sequence similar to the known Crp-binding sequence (TGTGA-N_6_-TCACA). Based on these findings, we assumed that Crp1 is not directly involved in the regulation of the *furA1-katG1* operon and that FurA is likely involved in upregulation of the operon in the Δ*crp1* mutant. Since FurA serves as a repressor for the regulation of the *furA1-katG1* operon and its functionality is inactivated by ROS such as H_2_O_2_ (36), we assessed whether intracellular ROS levels are increased in the Δ*crp1* mutant compared to the WT strain. As shown in Fig. 5A, ROS levels were increased by twofold in the Δ*crp1* mutant compared to the WT strain when both strains were grown aerobically. The complemented Δ*crp1* mutant harboring pMV306crp showed reduced ROS levels. These results indicate that the inactivation of Crp1 results in an increase in intracellular ROS levels when *M. smegmatis* is grown aerobically.

The respiratory ETC is a major source of ROS in aerobically growing bacteria and respiring mitochondria (51–56). Therefore, we measured the oxygen consumption rate using membrane fractions to examine whether the electron flow through the respiratory ETC is altered in the Δ*crp1* mutant compared to the WT strain. As shown in Fig. 5B, the oxygen consumption rate in the aerobically grown Δ*crp1* mutant harboring pMV306 was decreased relative to the WT strain with pMV306 grown under the same conditions, when either NADH or succinate was used as the electron donor. Introduction of *crp1* into the mutant restored the oxygen consumption rate to that of the WT strain, indicating that the inactivation of Crp1 leads to decreased electron flow through the respiratory ETC. The oxygen consumption assay also showed that NADH is a more efficient electron donor than succinate when both were used at the same concentration in the assay, suggesting that NDH serves as the major entry point of electrons in the respiratory ETC of *M. smegmatis*.

To examine whether the decreased respiration rate affects intracellular ATP levels in the Δ*crp1* mutant, we measured ATP concentrations in the WT and ∆*crp1* strains. Consistent with the reduced respiration, intracellular ATP levels were slightly lower in the ∆*crp1* mutant than those in the WT strain, with a decrease proportional to the reduction in the respiration rate (Fig. 5C). Taken together, the results presented in Fig. 5 indicate that the absence of Crp1 leads to increased ROS production, diminished electron flow through the respiratory ETC, and reduced ATP generation.

**Fig 6:**
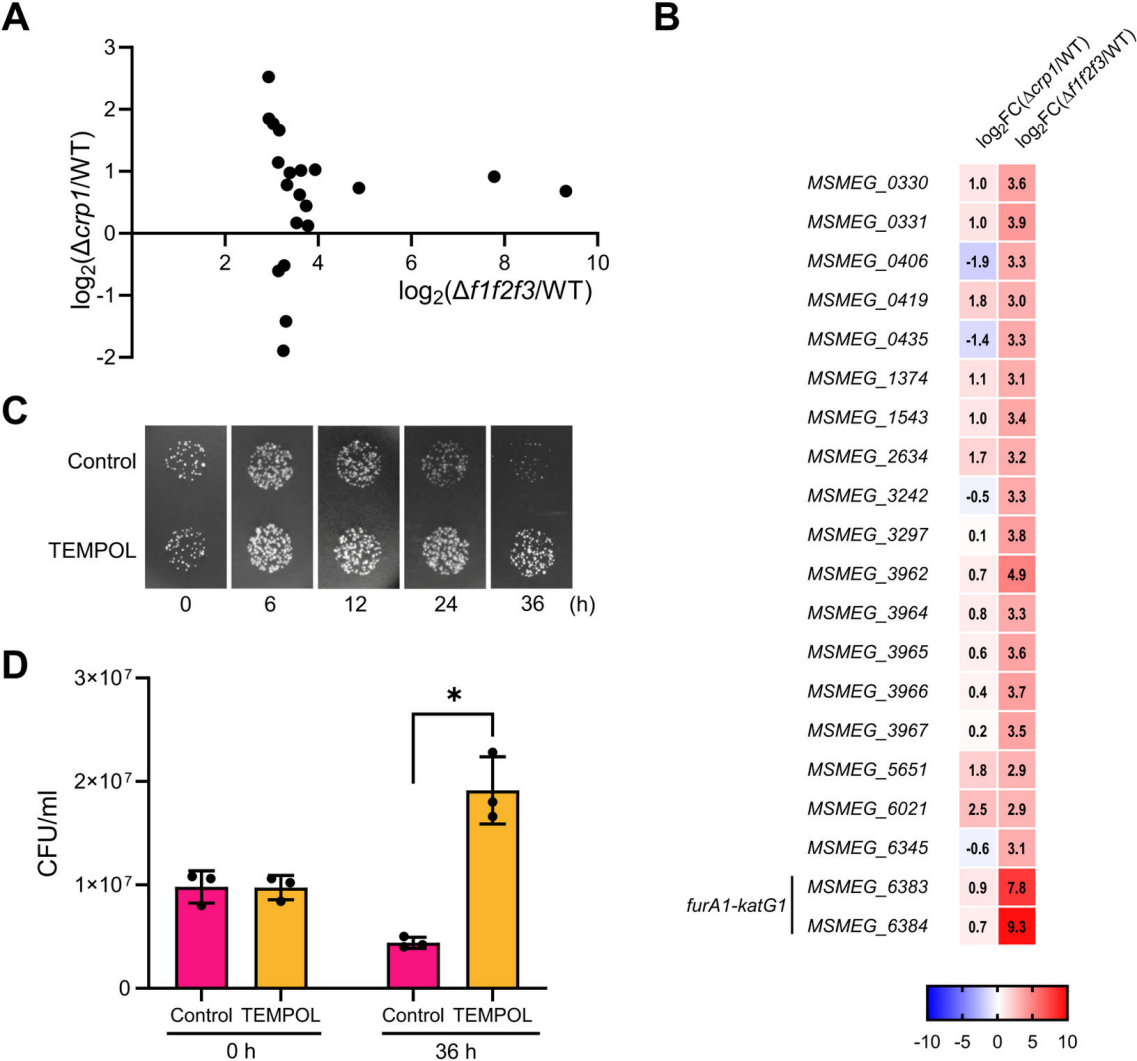
Correlation analysis showing induction of genes repressed by FurA in the Δ*crp1* mutant of *M. smegmatis* and the effect of TEMPOL on INH susceptibility of the Δ*crp1* mutant. (**A**) Scatter plot showing the expression levels of FurA-repressed differentially expressed genes (DEGs) in the Δ*f1f2f3* and Δ*crp1* mutants relative to the WT strain. The x-axis represents the log_2_ fold change (FC) in expression of the FurA-repressed DEGs in the Δ*f1f2f3* mutant relative to the WT strain, while the y-axis represents the log_2_ FC in expression of the DEGs in the Δ*crp1* mutant relative to the WT strain. Filled circles indicate the selected FurA-repressed DEGs, defined as the 20 most upregulated genes in the Δ*f1f2f3* mutant relative to the WT strain. The *ahpC*, *ahpD*, and *furA3* (*MSMEG_6253*) genes were excluded from the correlation analysis since Crp1 directly regulates the activity of *ahpCD* operon and *furA3* (37). The *furA2* and *katG2* genes were also excluded as the expression of the *furA2-katG2* operon has been shown not to be induced by ROS treatment (36). (**B**) The heatmap showing the locus tags and relative expression levels of the 20 FurA DEGs in the Δ*f1f2f3* and Δ*crp1* mutants relative to the WT strain. Transcriptomic data for the WT and mutant strains, grown aerobically to an OD_600_ of 0.45–0.5 in 7H9-glucose medium (35, 36), were obtained from NCBI’s Gene Expression Omnibus (https://www.ncbi.nlm.nih.gov/geo/) using the following accession numbers: GSE97620 (Δ*f1f2f3* mutant) and GSE158137 (Δ*crp1* mutant). (**C and D**) Spotting assay and CFU counting showing the effect of TEMPOL treatment on INH susceptibility of the Δ*crp1* strain. The Δ*crp1* strain was grown aerobically to an OD_600_ of 0.4, treated with INH at a final concentration of 20 µg/mL, and further incubated for up to 36 h. To examine the effect of TEMPOL, 5 mM TEMPOL was added to the cultures concurrently with INH. As a control, the same volume of distilled water (matching the TEMPOL stock solution) was added. At the indicated time points, 50 µL of cultures diluted 10^4^-fold was spotted onto 7H9-glucose agar plates lacking INH. The plates were incubated at 37°C for 3 days. For CFU counting, cultures collected at 0 and 36 h were used. CFU values were expressed as CFU per mL of culture. *P < 0.05.

Using the available RNA sequencing data for *M. smegmatis* (35, 36), a correlation plot was generated to examine whether genes repressed by FurA are upregulated in the Δ*crp1* mutant, similar to *furA1* and *katG1*. As shown in Fig. 6A and B, 16 of the 20 most highly derepressed genes in the *furA1furA2furA3* triple mutant (Δ*f1f2f3*) relative to the WT strain were upregulated in the Δ*crp1* mutant compared to the WT strain. The remaining four genes in the lower quadrant, which were downregulated in the Δ*crp1* mutant compared to the WT strain, may be under the direct positive regulation of Crp1, in addition to repression by FurA. This analysis suggests that elevated intracellular ROS levels in the Δ*crp1* mutant likely inactivate the FurA repressor, thereby leading to elevated expression of the FurA regulon, which includes the *furA1-katG1* operon.

We next examined whether treatment of the Δ*crp1* mutant with the ROS scavenger TEMPOL (4-hydroxy-2,2,6,6-tetramethylpiperidine-*N*-oxyl) could mitigate its increased susceptibility to INH. TEMPOL is known to efficiently remove ROS, including superoxide radicals and hydrogen peroxide (57). As determined by a spotting assay, co-treatment of INH and TEMPOL protected the Δ*crp1* mutant from bactericidal activity of INH, compared to INH treatment alone at a final concentration of 20 µg/mL (Fig. 6C). The results of colony-forming unit (CFU) counting were consistent with those of the spotting assay (Fig. 6D). These results indicate that elevated intracellular ROS levels in the Δ*crp1* mutant contribute, at least in part, to its increased susceptibility to INH.

### Crp1 inactivation leads to changes in oxidative phosphorylation

**Fig 7:**
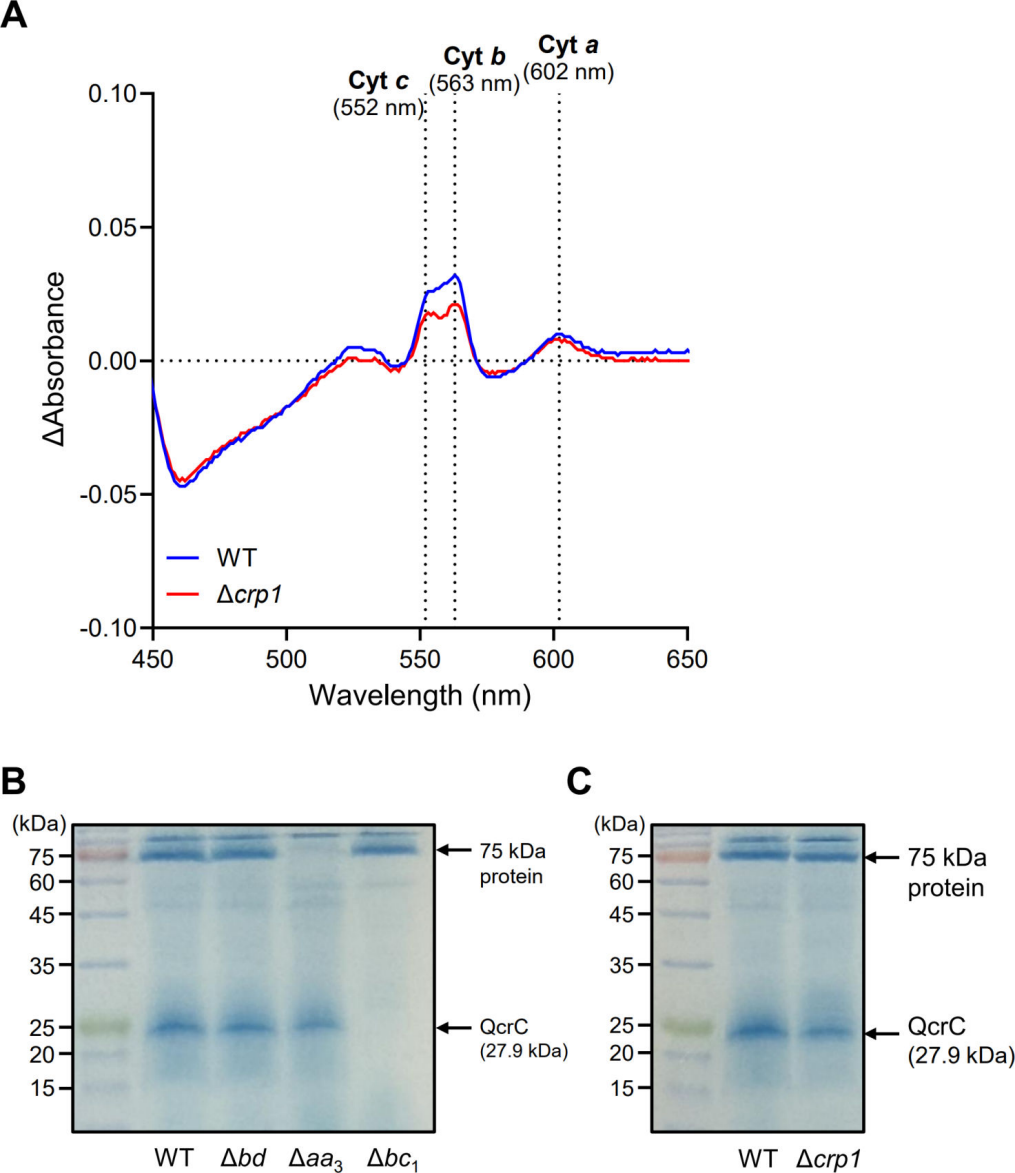
Quantitative comparison of cytochrome *bcc*_1_ complex and *aa*_3_ cytochrome *c* oxidase levels in membrane fractions from the WT and Δ*crp1* strains of *M. smegmatis*. (**A**) Dithionite-reduced minus ferricyanide-oxidized difference spectra recorded between 450 and 650 nm. Solubilized membrane proteins (2 mg/mL) from the WT (blue) and Δ*crp1* (red) strains were analyzed. Peaks at 552, 563, and 602 nm represent *c*-type, *b*-type, and *a*-type cytochromes, respectively. (**B**) Heme staining of solubilized membrane proteins (60 µg) from the WT, Δ*bd*, Δ*aa*_3_, and Δ*bc*_1_ strains. (**C**) Heme staining of solubilized membrane proteins (60 µg) from the WT and Δ*crp1* strains. The diheme cytochrome *cc*_1_ (QcrC) of the cytochrome *bcc*_1_ complex and a 75 kDa protein absent in the Δ*aa*_3_ mutant are indicated by arrows.

We observed reduced respiration and ATP levels, along with elevated ROS levels, in the Δ*crp1* mutant compared to the WT strain. These findings suggest that alterations in the respiratory ETC may affect ROS production in the Δ*crp1* mutant. To examine this hypothesis, we examined the levels of the cytochrome *bcc*_1_ complex and the *aa*_3_ cytochrome *c* oxidase by reduced-oxidized difference spectra and heme staining using membrane fractions from both WT and Δ*crp1* strains. As shown in Fig. 7A, the difference spectra revealed reduced α-band signals at 552 nm and 563 nm, corresponding to cytochrome *c* and cytochrome *b*, respectively, in the Δ*crp1* mutant compared to the WT strain. In contrast, the α-band at 602 nm representing cytochrome *a* was similar in magnitude in both WT and Δ*crp1* strains. Heme *c* serves as a cofactor of the diheme cytochrome *cc*_1_ (QcrC) in the *bcc*_1_ complex. Heme *b* is present in QcrB of the *bcc*_1_ complex, CydA of the cytochrome *bd* quinol oxidase, and Sdh2D of SDH2, while the CtaD subunit of the *aa*_3_ oxidase contains two *a*-type hemes (41, 49, 58). To confirm the reduced levels of cytochrome *c* in the mutant, we performed heme staining analysis, which specifically detects proteins with covalently bound hemes such as cytochrome *c*. Heme staining of the SDS-PAGE gel, in which solubilized membrane fractions from the WT, Δ*bd*, Δ*aa_3_*, and Δ*bc_1_* strains were separated, revealed two prominent bands: one corresponding to the diheme cytochrome *cc*_1_ (QcrC; 27.9 kDa) of the *bcc*_1_ complex and another at 75 kDa that was absent in the Δ*aa_3_* mutant (Fig. 7B). Consistent with the difference spectra presented in Fig. 7A, the ∆*crp1* mutant exhibited reduced levels of QcrC compared to the WT strain, while the levels of the 75 kDa protein were almost unchanged between the WT and ∆*crp1* strains (Fig. 7C). Taken together, these results indicate that the cytochrome *bcc*_1_ complex is present at reduced levels in the ∆*crp1* mutant relative to the WT strain, whereas the abundance of the *aa*_3_ cytochrome *c* oxidase remains unaffected.

**Fig 8:**
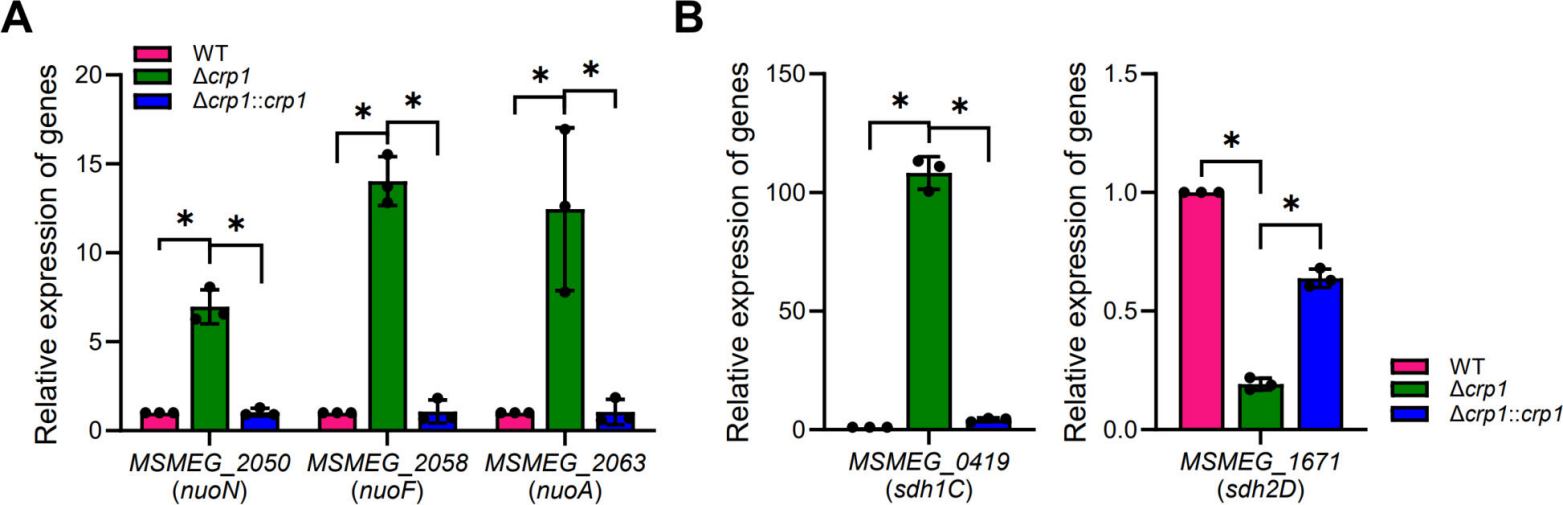
Expression levels of the *nuo*, *sdh1*, and *sdh2* in the WT and Δ*crp1* strains of *M. smegmatis*. (**A**) Expression levels of three representative genes (*MSMEG_2050*, *MSMEG_2058*, and *MSMEG_2063*) from the *nuo* operon encoding type I NDH in the WT and Δ*crp1* strains carrying pMV306, as well as in the complemented Δ*crp1::crp1* strain. (**B**) Expression levels of *MSMEG_0419* and *MSMEG_1671*, which belong to the *sdh1* and *sdh2* operons, respectively, in the WT and Δ*crp1* strains with pMV306, as well as in the Δ*crp1::crp1* strain. Expression levels of the genes were quantified by qRT-PCR and normalized to *sigA* expression. The expression level of each gene in the WT strain was set to 1, and the relative values were calculated for the other strains. All values represent the averages of three biological replicates, with error bars indicating standard deviations. *P < 0.05.

Flavin-containing NDH and SDH are known sites of ROS production in the respiratory ETC in addition to the *bc*_1_ complex (51). As shown in Fig. 2A, the expression of *ndh*, which encodes the major type II NDH, was unchanged in the Δ*crp1* mutant compared to the WT strain. In contrast, qRT-PCR analysis showed that the expressions of *nuoN* (*MSMEG_2050*), *nuoF* (*MSMEG_2058*), and *nuoA* (*MSMEG_2063*), which are components of the *nuo* operon encoding type I NDH, were increased by 7- to 14-fold in the Δ*crp1* mutant relative to the WT strain. This upregulation was reversed by complementation through the introduction of the intact *crp1* gene into the mutant (Fig. 8A). We examined the expressions of the *sdh1* and *sdh2* operons, which encode two isoforms of SDH. The expression of *sdh1C* (*MSMEG_0419*) from the *sdh1* operon was approximately 100-fold derepressed in the ∆*crp1* mutant compared to the WT strain, whereas the expression of *sdh2D* (*MSMEG_1671*) from the *sdh2* operon was reduced to approximately 20% of the WT level in the mutant. Complementation with the *crp1* gene restored the expression of both operons to WT levels (Fig. 8B). When inferred from the RNA sequencing data (35), the transcript levels of *nuoN* (8.3), *nuoF* (31), and *nuoA* (2.7) in the WT strain of *M. smegmatis* grown aerobically in 7H9-glucose medium were significantly lower than that of *ndh* (616), based on the reads per kilobase of transcript per million mapped reads (RPKM) values (the average of three replicates is shown in parentheses). This finding is consistent with the established role of type II NDH as the major NDH in *M. smegmatis* (19, 20). For SDH, the mRNA level of *sdh1C* (13.3) was much lower than that of *sdh2D* (367.3) in the WT strain, indicating that SDH2 is the predominantly expressed SDH in the WT strain under these conditions. Based on these findings, we assumed that the strong derepression of the *nuo* and *sdh1* operons in the Δ*crp1* mutant does not necessarily lead to a drastic increase in total NDH and SDH activities compared to the WT strain.

Our findings suggest that the decreased level of the cytochrome *bcc*_1_ complex in the Δ*crp1* mutant creates a bottleneck in the respiratory ETC. This bottleneck diminishes electron flow and reduces the oxygen consumption rate, despite the increased expression of the *nuo* and *sdh1* operons.

## DISCUSSION

Crp1 has been identified as the predominant Crp in *M. smegmatis*, based on comparative analyses of Crp1 and Crp2 at both RNA and protein levels (35). Although Crp1 and Crp2 recognize the same target DNA sequence (TGTGA-N_6_-TCACA), they exhibit several distinct biochemical properties. Crp2 binds cAMP with approximately 10-fold higher affinity than Crp1 (*K*_d_ = 3 µM vs ~30 µM, respectively), but exhibits weaker binding to the Crp consensus sequence (33). Considering Crp2’s substantially lower cellular abundance, weaker DNA-binding ability, much higher cAMP affinity, and the high intracellular cAMP concentrations in mycobacteria (35, 59–62), Crp1 is likely the principal regulator of cAMP-responsive gene expression in *M. smegmatis*. Consistent with this notion, altered INH susceptibility was observed in the Δ*crp1* mutant but not in the Δ*crp2* mutant.

Our Δ*crp1* mutant showed strong derepression of the *nuo* and *sdh1* operons but reduced expression of the *sdh2* operon compared to the WT strain, suggesting that Crp1 likely functions as a repressor of the *nuo* and *sdh1* operons and as an activator of the *sdh2* operon. Although less pronounced, similar regulation patterns of the *nuo*, *sdh1*, and *sdh2* operons by Crp2 (MSMEG_0539) have also been reported (34). It has been reported that the expressions of the *sdh1* and *sdh2* operons are regulated differently. The *sdh1* operon is significantly induced in *M. smegmatis* under energy-limiting (starvation) conditions but is repressed under hypoxic conditions, similar to the *nuo* operon. In contrast, the expression of the *sdh2* operon is upregulated under hypoxic conditions, with SDH2 playing an essential role under such conditions (48, 63). It has also been shown that intracellular cAMP levels are markedly elevated in *M. smegmatis* exposed to respiration-inhibitory conditions, such as hypoxia or inhibition of the *bcc*_1_-*aa*_3_ branch of the ETC (35, 64). By integrating these previous reports with our findings, we assumed that under energy-limiting conditions, depletion of intracellular ATP leads to a decrease in cAMP levels, resulting in less transcriptional activity of Crp1, whereas under respiration-inhibitory conditions such as hypoxia, intracellular cAMP levels are increased, allowing Crp1 to function as an active regulator.

We found that the cellular levels of the cytochrome *bcc*_1_ complex were reduced in the Δ*crp1* mutant relative to the WT strain, while the levels of the *aa*_3_ cytochrome *c* oxidase remained unchanged in the mutant. It has been reported that the *cydAB* operon encoding the cytochrome *bd* quinol oxidase is strongly induced in a Crp1-dependent manner under respiration-inhibitory conditions, such as hypoxia or inhibition of the *bcc*_1_-*aa*_3_ branch of the respiratory ETC (30, 35). Taken together, these findings indicate that Crp1 plays a key role in regulating oxidative phosphorylation in *M. smegmatis* by reflecting intracellular energy status and the functional state of the respiratory ETC.

We demonstrated that ROS levels were elevated in the Δ*crp1* mutant compared to the WT strain. The observation that most genes strongly repressed by FurA were derepressed in the mutant further supports the finding that ROS levels were increased in the Δ*crp1* mutant. Since the respiratory ETC is a major source of ROS generation, the increased ROS levels in the Δ*crp1* mutant may be attributable to alterations in ETC functionality. NDH (complex I), SDH (complex II), and cytochrome *bc*_1_ complex (complex III) are proposed sites of ROS production in the respiratory ETC. Electron leakage from reduced FMN or FAD and from reduced quinol to molecular oxygen during catalytic processes in complexes I, II, and III can lead to the formation of superoxide radicals and hydrogen peroxide (51–56, 65). In mycobacteria, the cytochrome *bcc*_1_ complex transfers electrons from menaquinol to the *aa*_3_ cytochrome *c* oxidase. The *bd* quinol oxidase also oxidizes menaquinol to menaquinone to reduce molecular oxygen. A decrease in cytochrome *bcc*_1_ complex abundance, accompanied by increased expressions of NDH and SDH, can create a bottleneck in electron flow through the *bcc*_1_-*aa*_3_ branch of the ETC. In the absence of a compensatory increase in cytochrome *bd* quinol oxidase levels, this bottleneck may prolong the lifetime of reduced flavin in NDH and SDH, shift the menaquinol/menaquinone pool toward a more reduced state upstream of the *bcc*_1_ complex, and promote reverse electron transport at complex I. Under these conditions, as observed in the Δ*crp1* mutant, electron leakage from reduced flavin and menaquinol to oxygen is likely enhanced, thereby promoting ROS formation.

**Fig 9:**
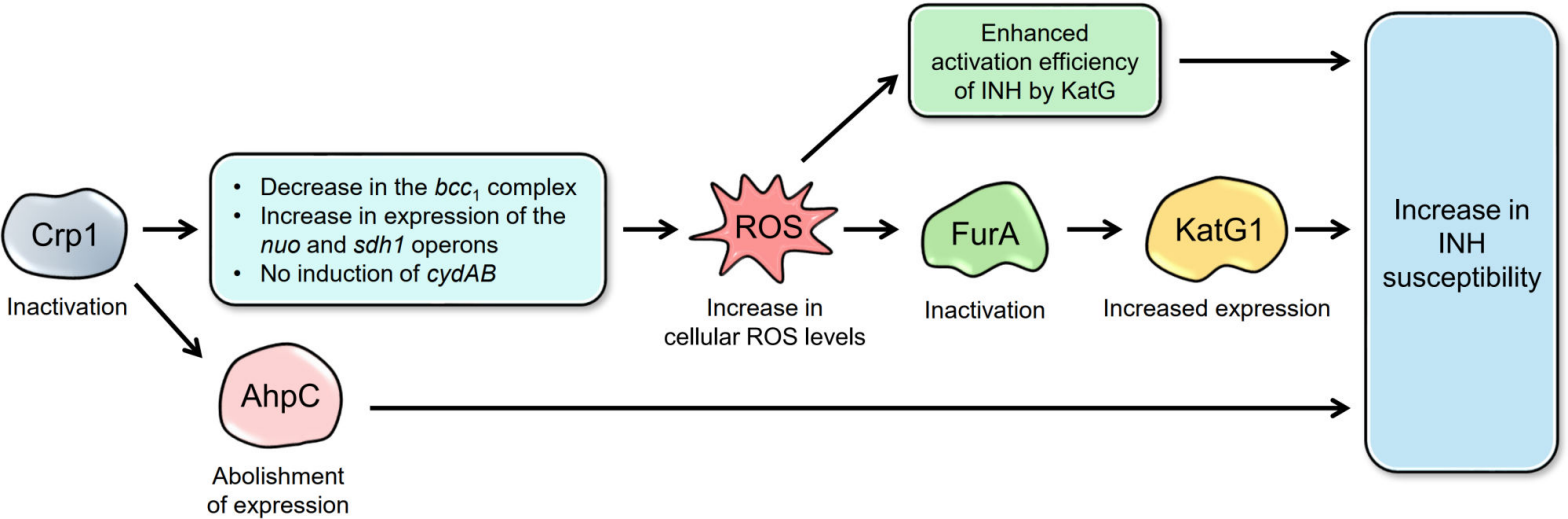
Proposed model for the mechanism underlying the increased sensitivity of the Δ*crp1* mutant to INH. Inactivation of Crp1 reduces the level of the cytochrome *bcc*_1_ complex and abolishes the induction of the *bd* quinol oxidase, while increasing the expressions of type I NDH and SDH1 in *M. smegmatis*. These alterations in respiratory ETC functionality lead to increased intracellular ROS levels, which inactivate FurA, a repressor of *katG1*. Loss of FurA repression results in upregulation of *katG1* expression. The combined effect of increased KatG1 activity and elevated ROS enhances INH activation, thereby increasing the susceptibility of the Δ*crp1* mutant to INH. In addition, Crp1 inactivation abolishes *ahpC* expression, further contributing to the increased INH sensitivity of the mutant.

The increased INH susceptibility of the ∆*crp1* mutant is most likely attributable to elevated intracellular ROS levels, as supported by both previous findings and our observations (Fig. 9). First, we found that ROS levels were higher in the ∆*crp1* mutant than in the WT strain. Elevated ROS can inactivate the FurA repressor, leading to the overproduction of the KatG1 catalase-peroxidase, the enzyme that activates the prodrug INH. The peroxidatic activity of KatG1, which converts INH into the isonicotinoyl radical, has been proposed to require H₂O₂ or simple alkyl hydroperoxides as oxidants (6–9). In addition, treatment with the ROS scavenger TEMPOL was shown to mitigate the enhanced INH susceptibility of the ∆*crp1* mutant (Fig. 6C).

In the ∆*crp1* mutant, the absence of *ahpC* expression further contributes to its increased INH susceptibility, in addition to the effects of elevated ROS levels (Fig. 2B). Because the deletion of the *ahpCD* operon did not increase *katG1* expression (Fig. 3D), the elevated ROS levels in the ∆*crp1* strain are unlikely to result from the loss of alkyl hydroperoxide reductase. This also indicates that the increased INH susceptibility observed in the ∆*ahpCD* mutant is not caused by KatG1 overproduction due to the loss of alkyl hydroperoxide reductase. Thus, the precise mechanism by which alkyl hydroperoxide reductase affects INH susceptibility remains elusive.

Overall, our findings highlight that the integrity of the respiratory ETC influences INH susceptibility in *M. smegmatis*, likely through its effects on ROS levels, the NADH/NAD^+^ ratio, and other interconnected cellular processes.

In conclusion, we found that the inactivation of *crp1* markedly increases the susceptibility of *M. smegmatis* to INH, but not to RIF. This increased INH susceptibility arises from multiple factors, including the elevated expression of the INH-activating catalase-peroxidase (KatG1), the abolishment of *ahpC* expression, and increased cellular ROS levels. The increase in ROS levels and the associated upregulation of *katG1* appear to be driven by alterations in the respiratory ETC function, including reduced levels of the cytochrome *bcc*_1_ complex, upregulation of the expressions of *nuo* and *sdh1* operons, and the loss of *bd* quinol oxidase induction under conditions of diminished *bcc*_1_ complex abundance.

## MATERIALS AND METHODS

### Bacterial strains, plasmids, and culture conditions

The bacterial strains and plasmids used in this study are listed in Table 1. *Escherichia coli* strains were cultivated in lysogeny broth (LB) medium on a gyratory shaker (200 rpm) at 37°C. *M. smegmatis* strains were grown in Middlebrook 7H9 medium (Difco, Sparks, MD) supplemented with 0.2% (wt/vol) glucose (7H9-glucose). Tween 80 was added to growth medium for *M. smegmatis* at a final concentration of 0.02% (vol/vol) as an anti-clumping agent. *M. smegmatis* strains were grown aerobically on a gyratory shaker (200 rpm) to an OD_600_ of 0.45 to 0.5 at 37°C. Kanamycin (50 µg/mL for *E. coli* and 15 or 30 µg/mL for *M. smegmatis*) and hygromycin (200 µg/mL for *E. coli* and 25 or 50 µg/mL for *M. smegmatis*) were added to the growth medium when required. The cloned gene under the control of an acetamide-inducible promoter on pMH201 derivatives was overexpressed in *M. smegmatis* by inducing with 0.1% (wt/vol) acetamide.

**TABLE 1:** Strains and plasmids used in this study

Strain/plasmid	Relevant phenotype/genotype^*a*^	Reference
Strains		
*E .coli* DH5α	φ80d*lacZ*ΔM15 Δ*lacU169 recA1 endA1 hsdR17 supE44 thi1 gyrA96 relA1*	(66)
*M. smegmatis* mc^2^155	High-transformation-efficiency mutant of *M. smegmatis* ATCC 607	(67)
*M. smegmatis* Δ*crp1*	*MSMEG_6189* (*crp1*) deletion/insertion mutant derived from *M. smegmatis* mc^2^155; Hyg^r^; Previously, this mutant was named *crp*. To distinguish *crp1* and *crp2* mutants, we renamed the *crp* mutant to Δ*crp1*	(37)
*M. smegmatis* Δ*crp2*	*MSMEG_0539* (*crp2*) deletion mutant derived from *M. smegmatis* mc^2^155	(35)
*M. smegmatis* Δ*ahpCD*	*MSMEG_4891* (*ahpC*) - *MSMEG_4890* (*ahpD*) operon deletion mutant derived from *M. smegmatis* mc^2^155	This study
*M. smegmatis* Δ*katG1*	*MSMEG_6384* (*katG1*) deletion mutant derived from *M. smegmatis* mc^2^155	This study
*M. smegmatis* Δ*katG2*	*MSMEG_3461* (*katG2*) deletion mutant derived from *M. smegmatis* mc^2^155	This study
*M. smegmatis* Δ*katG12*	*MSMEG_6384* (*katG1*) and *MSMEG_3461* (*katG2*) double-deletion mutant derived from *M. smegmatis* mc^2^155	This study
*M. smegmatis* Δ*bd*	*MSMEG_3233* (*cydA*) deletion mutant derived from *M. smegmatis* mc^2^155	(43)
*M. smegmatis* Δ*aa*_3_	*MSMEG_4268* (*ctaC*) deletion mutant derived from *M. smegmatis* mc^2^155	(43)
*M. smegmatis* Δ*bc*_1_	*MSMEG_4261* (*qcrC*)*, MSMEG_4262* (*qcrA*), and *MSMEG_4263* (*qcrB*) deletion mutant derived from *M. smegmatis* mc^2^155	(68)
Plasmids		
pKOTs	Hyg^r^; pKO-based vector constructed by inserting the HindIII-KpnI fragment containing pAL500Ts and pUC ori derived from pDE	(69)
pMV306	Km^r^; integration vector containing *int* and the *attP* site of mycobacteriophage L5 for integration into the mycobacterial genome	(70)
pMH201	Km^r^; acetamide-inducible promoter, derivative of pMV306	(71)
pEM	Km^r^; promoterless lacZ	This study
pEMfurA1	pEM::0.489 kb XbaI-ClaI fragment containing the *furA1* promoter region	This study
pEMSD1	pEM::0.287 kb XbaI-ClaI fragment containing the *furA1* promoter region	This study
pEMSD2	pEM::0.188 kb XbaI-ClaI fragment containing the *furA1* promoter region	This study
pEMSD3	pEM::0.098 kb XbaI-ClaI fragment containing the *furA1* promoter region	This study
pEMSD4	pEM::0.048 kb XbaI-ClaI fragment containing the *furA1* promoter region	This study
pMV306crp1	pMV306::1.239 kb ClaI-HindIII fragment containing *crp1*	(37)
pMH201katG1	pMH201::2.255 kb NdeI-ClaI fragment containing *katG1*	This study
pKOTsΔahpCD	pKOTs::0.672 kb NotI-HindIII fragment containing Δ*ahpCD*	This study
pKOTΔkatG1	pKOTs::0.631 kb DNA fragment containing Δ*katG1*	This study
pKOTΔkatG2	pKOTs::0.668 kb DNA fragment containing Δ*katG2*	This study

^*a*^ Hyg^r^, hygromycin resistance; Km^r^, kanamycin resistance.

### DNA manipulation and transformation

Standard protocols and manufacturers’ instructions were followed for recombinant DNA manipulations (72). Transformation of *M. smegmatis* with plasmids was carried out by electroporation as previously described (67). The primers used for PCR are listed in Table 2.

**TABLE 2:** Oligonucleotides used in this study

Oligonucleotide	Nucleotide sequence (5'→3')	Purpose
F_ahpCDmut	ATTTGCGGCCGCAAGGATTACGAGGGCAAG	Δ*ahpCD* construction
R_ahpCDmut	ATTTAAGCTTGGGCAGTGGTCGTAC	Δ*ahpCD* construction
F_ahpCDrec	GGTCCAGTTCGTGTCGGTGGCGATCATGGGGATGAACAAC	Δ*ahpCD* construction
R_ahpCDrec	GTTGTTCATCCCCATGATCGCCACCGACACGAACTGGACC	Δ*ahpCD* construction
F_katG1mut	ATTTGCGGCCGCAAGTACGAGGAGATCC	Δ*katG1* and Δ*katG12* construction
R_katG1mut	ATTTAAGCTTTGCGCATCTGTTCCAC	Δ*katG1* and Δ*katG12* construction
F_katG1rec	CTGAGATGACCACGCTCGTCGACACACAACCACCTGGAC	Δ*katG1* and Δ*katG12* construction
R_katG1rec	GTCCAGGTGGTTGTGTGTCGACGAGCGTGGTCATCTCAG	Δ*katG1* and Δ*katG12* construction
F_katG2mut	ATTTGCGGCCGCAGGACTTCAACGCCTC	Δ*katG2* and Δ*katG12* construction
R_katG2mut	ATTTAAGCTTCCAGGGTTGCGTATTCG	Δ*katG2* and Δ*katG12* construction
F_katG2rec	CGCTGGACGTCAACCACGGTAAACCGAGAACTGGTCAC	Δ*katG2* and Δ*katG12* construction
R_katG2rec	GTGACCAGTTCTCGGTTTACCGTGGTTGACGTCCAGCG	Δ*katG2* and Δ*katG12* construction
F_katG1ox	ATTTCATATGCCTGAGGATCGCCCGATC	*katG1* overexpression
R_katG1ox	ATTTATCGATTCAGTGATGGTGATGGTGATGGGCGACGTCGAAGCGGTC	*katG1* overexpression
F_furA1lacZ	ATATTCTAGATCGTGGTCCCCACCGAGGC	*furA1*::*lacZ* fusion
F_ furA1SD1	ATATTCTAGACAGGCGCGTACTGGAGTTC	*furA1*::*lacZ* fusion
F_ furA1SD2	ATATTCTAGAGATCAGGATCCGCCGCAGC	*furA1*::*lacZ* fusion
F_ furA1SD3	ATATTCTAGAGCGAGAACTTTATTCTTGAC	*furA1*::*lacZ* fusion
F_ furA1SD4	ATATTCTAGAGTGACCCACACGACCGACTTC	*furA1*::*lacZ* fusion
R_furA1lacZ	ATTTATCGATCATGCCGGCACTTCGAAG	*furA1*::*lacZ* fusion
F_sigA_RT	CTGGAGGCGAACCTGCGC	qRT-PCR
R_sigA_RT	CTGGTCGGCCATGGCGCG	qRT-PCR
F_katG1_RT	GTGGCCCAATCAGCTCAATC	qRT-PCR
R_katG1_RT	GATGAACAGCGGTCCGTAG	qRT-PCR
F_katG2_RT	AACCAGATCGACGTATCACGCC	qRT-PCR
R_katG2_RT	CAGCTCATACGGATGAACAGGC	qRT-PCR
F_ahpC_RT	GTGTGTCGGTGGACAACGAG	qRT-PCR
R_ahpC_RT	GGTCACCGACACGAACTGGA	qRT-PCR
F_inhA_RT	AAGCGCATCCTCGTCACGGG	qRT-PCR
R_inhA_RT	AGAGTCGACAGGTGCTCCTC	qRT-PCR
F_mspA_RT	CATCGCGGCGCTTTTCAC	qRT-PCR
R_mspA_RT	GCGGTCCTGGCCATCAAC	qRT-PCR
F_rbpA_RT	GGGGCAGTCGCCTCGGAG	qRT-PCR
R_rbpA_RT	AGCATGTCCCAGTGCGTACG	qRT-PCR
F_ndh_RT	GGTTCGGGTTTCGGTGGTCTC	qRT-PCR
R_ndh_RT	CGTTCTTCTGCTTGCGGAGGATC	qRT-PCR
F_mdp1_RT	GAGCTCATCGACGTACTCAC	qRT-PCR
R_mdp1_RT	GCTGCTCGAAGACACCGAAC	qRT-PCR
F_cydA_RT	CGGTGGCAGTTCGGAATCAC	qRT-PCR
R_cydA_RT	CAGAAAAAGTTTGCCGAAGAAACG	qRT-PCR
F_2050_RT	GACCGACGTCAAACGCATGCT	qRT-PCR
R_2050_RT	AGCGTGCTGAACCCGTAGGC	qRT-PCR
F_2058_RT	ATCGCCTGGCTTCACGCTGT	qRT-PCR
R_2058_RT	CCGCCAGGCGTCCAGAAC	qRT-PCR
F_2063_RT	GCCAGCGCATACCGATCCG	qRT-PCR
R_2063_RT	CCTCGCCGCCAGACATAGG	qRT-PCR
F_0419_RT	GCGTTCTGGGGCAGTGCG	qRT-PCR
R_0419_RT	CCAGTGTATTTCGCGTGCGGC	qRT-PCR
F_1671_RT	GGCGCCCGTGATGGAACGTG	qRT-PCR
R_1671_RT	CCAGCGCAATGCCGGAGAAC	qRT-PCR

### Construction of plasmids

#### pKOTsΔahpCD, pKOTsΔkatG1, and pKOTsΔkatG2

Temperature-sensitive suicide plasmids were used for the construction of mutant strains of *M. smegmatis*. To construct pKOTsΔahpCD, two rounds of recombination PCR were performed. Using the chromosomal DNA of *M. smegmatis* as a template, two primary PCRs were conducted. The first reaction utilized the primers F_ahpCDmut and R_ahpCDrec, while the second used the primers F_ahpCDrec and R_ahpCDmut. These reactions generated two overlapping DNA fragments of 360 bp and 352 bp, each containing a 40 bp overlap. Both PCR products contained the same 369 bp deletion within the *ahpC-ahpD* operon in their overlapping regions. In the secondary PCR, a 672 bp DNA fragment with a deletion in *ahpCD* was obtained using the primary PCR products as templates, along with the primers F_ahpCDmut and R_ahpCDmut. The resulting secondary PCR product was restricted with NotI and HindIII and cloned into pKOTs digested with the same enzymes, resulting in pKOTsΔahpCD.

To construct pKOTsΔkatG1, two rounds of recombination PCR were conducted. Using the chromosomal DNA of *M. smegmatis* as a template, two primary PCRs were performed with the primers F_katG1mut and R_katG1rec, and with the primers F_katG1rec and R_katG1mut, generating two overlapping DNA fragments (340 and 330 bp) with a 39 bp overlap. Both PCR products contained the same 340 bp deletion within *katG1* in their overlapping regions. In the secondary PCR, a 631 bp DNA fragment with a deletion of *katG1* was obtained using both primary PCR products as templates, along with the primers F_katG1mut and R_katG1mut. The secondary PCR product was restricted with NotI and HindIII and cloned into pKOTs, resulting in pKOTsΔkatG1.

To construct pKOTsΔkatG2, two rounds of recombination PCR were conducted. Using the chromosomal DNA of *M. smegmatis* as a template, two primary PCRs were performed. The first reaction used the primers F_katG2mut and R_ katG2rec, while the second used the primers F_ katG2rec and R_ katG2mut. These reactions generated two overlapping DNA fragments of 371 bp and 335 bp, each containing a 38 bp overlap. Both PCR products contained the same 307 bp deletion within *katG2* in their overlapping regions. In the secondary PCR, a 668 bp DNA fragment with a deletion of *katG2* was obtained using both primary PCR products as templates and the primers F_katG2mut and R_katG2mut. The secondary PCR product was restricted with NotI and HindIII and cloned into pKOTs, yielding pKOTsΔkatG2.

#### pEMfurA1, pEMSD1, pEMSD2, pEMSD3, and pEMSD4

The plasmid pEMfurA1 is a *furA1::lacZ* transcriptional fusion plasmid that contains the 5′ portion (48 bp) of *furA1*, along with a 441 bp DNA sequence upstream of *furA1*. To construct pEMfurA1, a 489 bp DNA fragment was amplified by using the chromosomal DNA of *M. smegmatis* as a template, along with the primers F_furA1lacZ and R_furA1lacZ. The PCR product was restricted with XbaI and ClaI and cloned into the pEM, yielding pEMfurA1.

The plasmids pEMSD1, pEMSD2, pEMSD3, and pEMSD4 are serially truncated derivatives of pEMfurA1 that contain the 5′ portion (48 bp) of *furA1* along with 239 bp, 140 bp, 50 bp, and 0 bp DNA sequences upstream of the *furA1* start codon, respectively. DNA fragments were amplified by PCR using the primers F_furA1SD1, F_furA1SD2, F_furA1SD3, or F_furA1SD4 in combination with R_furA1lac. The PCR products were digested with XbaI and ClaI and cloned into the pEM, resulting in pEMSD1, pEMSD2, pEMSD3, and pEMSD4.

#### pMH201katG1

To construct pMH201katG1 for *katG1* overexpression in *M. smegmatis*, a 2,255 bp DNA fragment containing the *katG1 gene* and six His codons immediately before its stop codon was amplified by PCR with the primers F_katG1ox and R_katG1ox using the chromosomal DNA of *M. smegmatis* as a template. The PCR product was restricted with NdeI and ClaI and cloned into the pMH201 integration vector with an acetamide-inducible promoter, generating pMH201katG1.

### Construction of mutant strains of *M. smegmatis*

The Δ*ahpCD*, Δ*katG1*, Δ*katG2*, and Δ*katG12* mutants of *M. smegmatis* were generated through allelic exchange mutagenesis, using the corresponding pKOTs-derived suicide plasmids (pKOTsΔahpCD, pKOTsΔkatG1, and pKOTsΔkatG2). The mutagenesis was performed in the background of the WT or Δ*katG1* strain, following the procedure previously described (69). In brief, the temperature-sensitive suicide plasmid was introduced into *M. smegmatis* by electroporation. Transformants were selected at 30°C (replication-permissive temperature) on 7H9-glucose agar plates containing hygromycin, and the selected transformants were grown in 7H9-glucose liquid medium supplemented with hygromycin for 3 days at 30°C. Heterogenotes of *M. smegmatis*, which were generated by a single recombination event, were selected for their hygromycin resistance on 7H9-glucose agar plates at 42°C (replication-nonpermissive temperature). The selected heterogenotes were grown on 7H9-glucose medium without antibiotics for 3 days at 37°C. Isogenic homogenotes were obtained from the heterogenotes after a second recombination by selecting them for sucrose resistance on 7H9-glucose agar plates containing 10% (wt/vol) sucrose at 37°C.

### Quantitative real-time PCR

RNA isolation from *M. smegmatis* strains and cDNA synthesis were performed as previously described (73), except that a random hexamer primer (Thermo Fisher Scientific, Waltham, MA) was used instead of gene-specific primers in cDNA synthesis. The isolated RNA was assessed for DNA contamination by PCR using the primers designed for quantitative real-time PCR (qRT-PCR). qRT-PCR was performed to determine the transcript levels of *katG1*, *katG2*, *ahpC*, *inhA*, *mspA*, *rbpA*, *ndh*, *mdp1*, *cydA*, *nuoA*, *nuoF*, *nuoN*, *sdh1C*, and *sdh2D*. The reaction was conducted in a 20 µL mixture containing 5 µL of template cDNA, 1 µL (15 pmol) of each gene-specific primer, 10 µL of TB Green Premix Ex Taq (Tli RNase Plus) (Takara, Tokyo, Japan), 0.4 µL of ROX passive fluorescent dye, and 2.6 µL of distilled water. The thermal cycling process began with an initial denaturation step at 95°C for 2 min, followed by 40 cycles of 95°C for 5 sec and 64°C for 30 sec. The *sigA* gene, encoding the principal sigma factor, was used as a reference gene for qRT-PCR to normalize the expression levels of the tested genes since our RNA sequencing analyses revealed that the *sigA* gene is constitutively expressed at similar levels in the WT and Δ*crp1* strains (35). Melting curve analysis was performed for each reaction to confirm the amplification of a single PCR product. The primers used for qRT-PCR are listed in Table 2.

### Zone inhibition assay

*M. smegmatis* strains were cultivated aerobically in 7H9-glucose medium until the OD_600_ reached 0.45–0.5. Five mL of culture was spread uniformly onto 7H9-glucose agar plates, and the excess culture was drained off. The plates were tapped on a paper towel to remove any excess liquid. They were then dried at room temperature for 2 to 3 h. A paper disk was placed onto each dried plate, and 10 µL of the specified concentrations of INH or RIF was applied to the disk. The plates were incubated at 37°C for 3 days to observe zones of growth inhibition.

### Determination of minimum inhibitory concentration (MIC)

*M. smegmatis* strains were aerobically cultivated in 7H9-glucose medium supplemented with 0.2% (vol/vol) glycerol and 0.02% (vol/vol) Tween 80 until the OD_600_ reached 0.5–1.0. The harvested cells were washed twice with 7H9 medium supplemented with 0.05% (vol/vol) Tween 80 and resuspended in 7H9-glucose medium supplemented with 0.2% (vol/vol) glycerol and 0.05% (vol/vol) Tween 80 to an OD_600_ of 0.1. A 1.5 mL aliquot of the cell suspension was placed into the wells of 12-well plates, each containing a ceramic ball with a diameter of 6.3 mm for agitation. After treating the *M. smegmatis* cultures with INH or RIF at specified concentrations, the strains were further incubated for 24 h (WT, Δ*crp2*, and complemented Δ*crp1* strains) or 42 h (Δ*crp1* mutant) at 37°C on a gyratory shaker at 100 rpm. Resazurin was added to the cultures to a final concentration of 6.25 µg/mL, and the cultures were further incubated for 3 h. The optical density of samples grown in the 12-well plates was measured at 570 nm using a BioTek Synergy H1 plate reader (Agilent, Santa Clara, CA).

### Spotting assay and CFU counting for determining INH susceptibility

The Δ*crp1* mutant of *M. smegmatis* was grown aerobically to an OD_600_ of 0.4 in 7H9-glucose medium and then treated with INH at a final concentration of 20 µg/mL for up to 36 h. At the indicated time points, 50 µL of cultures diluted 10^4^-fold with 7H9 medium was spotted onto 7H9-glucose agar plates lacking INH. For CFU counting, cultures collected at 0 and 36 h were serially diluted with 7H9 medium and spread onto 7H9-glucose agar plates without INH. The plates were incubated at 37°C for 3 days, and colonies were then counted. CFU values were expressed as CFU per mL of culture.

### β-Galactosidase assay and protein quantitation

β-Galactosidase activity was measured spectrophotometrically as described previously (74). Protein concentration was determined using the Bio-Rad protein assay kit (Bio-Rad, Hercules, CA) with bovine serum albumin (BSA) as the standard protein.

### Peroxidase activity staining

Peroxidase activity staining was conducted using the method described by Wayne and Diaz (75). Briefly, harvested cells were resuspended in 20 mM Tris-HCl buffer (pH 8.0) and disrupted by passage through a French pressure cell. Cell-free crude extracts were obtained by centrifugation at 19,000 × *g* for 10 min at 4°C to remove unbroken cells and cell debris. Crude extracts (25 µg) from *M. smegmatis* strains were subjected to native PAGE on a 7.5% (wt/vol) polyacrylamide gel. After electrophoresis, the gel was washed three times for 15 min with PBS and then incubated in 100 mL of PBS containing 50 mg of 3,3-diaminobenzidine and 10 µL of 30% (wt/vol) H_2_O_2_ with gentle shaking for 40 min in the dark. The staining solution was discarded, and the gel was rinsed with distilled water.

### Determination of intracellular ROS levels

Intracellular ROS levels were determined using the fluorescence-based dye 2’,7’-dichlorofluorescein diacetate (DCF-DA). *M. smegmatis* strains were aerobically grown to an OD_600_ of 0.45–0.5 in 7H9-glucose medium. Harvested cells were washed twice with 7H9 medium supplemented with 0.02% (vol/vol) Tween 80 and resuspended in 7H9-glucose medium supplemented with 0.02% (vol/vol) Tween 80 to an OD_600_ of 0.5. A 2 mL aliquot of the cell suspension was added to the wells of a 6-well plate and treated with DCF-DA to a final concentration of 10 µM for 30 min. To remove extracellular DCF-DA, cells harvested from 1 mL of the cell suspension were resuspended in 1 mL of PBS. The fluorescence intensity of DCF was measured using a BioTek Synergy H1 plate reader (Agilent, Santa Clara, CA) with an excitation/emission filter set at 488/525 nm.

### Quantitation of cellular ATP levels

*M. smegmatis* strains were grown aerobically to an OD_600_ of 0.45–0.5 at 37°C in 7H9-glucose medium. Cultures were harvested by centrifugation and resuspended in an equal volume of Tris-EDTA buffer (100  mM Tris [pH 7.75], 4  mM EDTA). Cells were disrupted three times using a Fastprep-24 5G (MP Biomedicals, Irvine, CA), and cell-free crude extracts were obtained by centrifugation at 19,000 × *g* for 5 min at 4°C. Protein concentration was determined, and ATP levels in the crude extracts were quantified using an ATP bioluminescence assay kit HSII (Roche, Basel, Switzerland) following the manufacturer’s instruction. Briefly, crude extracts were heated at 100°C for 5  min and cooled on ice for 2 min and centrifuged at 19,000 × *g* for 5 min at 4°C to remove denatured proteins. Aliquots (50 µL) of the prepared samples or ATP standard solutions were loaded into a black microtiter plate, and the reaction was started by adding 50 µL of luciferase reagent. Bioluminescence was measured for 1 to 10 s after a 1 s delay using a Mithras LB 940 luminometer (Berthold, Bad Wildbad, Germany). The levels of ATP in the samples were calculated from a standard curve generated using ATP standard solutions and normalized to the protein concentration.

### Preparation of membrane fractions and solubilized membrane proteins

Harvested cells of *M. smegmatis* were resuspended in buffer A (20 mM Tris-HCl, pH 7.5) and disrupted by five passages through a French pressure cell. Cell-free crude extracts were obtained by centrifugation twice at 27,000 × *g* for 15 min at 4°C. Membrane fractions were from the crude extracts by ultracentrifugation at 150,000 × *g* for 90 min at 4°C. The membrane pellets were washed twice with buffer A, solubilized in buffer A containing 0.5% (wt/vol) sodium dodecyl maltoside by pipetting, and centrifuged at 16,000 × *g* for 10 min at 4°C. The supernatant was collected as solubilized membrane proteins.

### Reduced minus oxidized difference spectrum analysis

Solubilized membrane proteins (2 mg/mL) were oxidized with 100 µM potassium ferricyanide for 2 min at room temperature, and then the oxidized spectrum was recorded using a UV 1601PC spectrophotometer (Shimadzu Corp., Columbia, MD). An equivalent amount of solubilized membrane proteins was reduced with 30 µL of 15% (wt/vol) sodium dithionite for 2 min at room temperature, and the reduced spectrum was recorded. The dithionite-reduced minus ferricyanide-oxidized spectrum was obtained by subtracting the two spectra. The amounts of *b*- and *c*-type hemes were estimated using the extinction coefficients ε_562–577_ of 22 mM^−1^ cm^−1^ and ε_552–540_ of 19.1 mM^−1^ cm^−1^, respectively.

### Heme staining

Heme staining of proteins with covalently bound heme was performed as described previously (76). After SDS-PAGE of solubilized membrane proteins, cytochromes were visualized via their intrinsic peroxidase activity using 3,3′,5,5′-tetramethyl benzidine and H_2_O_2_.

### Determination of the oxygen consumption rate

Prepared membrane fractions were washed twice and resuspended in 50 mM potassium phosphate (PP) buffer (pH 7.0). Oxygen consumption rates were determined using NADH or succinate as the electron donor. Measurements were performed polarographically using a YSI 5300 Clark-type electrode (Yellow Springs Instrument Co., Yellow Springs, OH), using 500 µg of membrane fractions in 5 mL of PP buffer. The measurement was initiated by adding NADH or succinate to a final concentration of 1 mM, and oxygen consumption was recorded for 5 min at 30°C.

### Statistical analysis

Statistical analysis was performed using GraphPad Prism 10.0 (GraphPad Software Inc). Each experiment included at least three biological replicates. Data were analyzed using an unpaired Student’s t test, and differences were considered statistically significant at *P* < 0.05.
